# Supplementary material for: Stimulus-choice (mis)alignment in primate area MT
Source: PLoS Comput Biol. 2020 May 18;16(5):e1007614. doi: 10.1371/journal.pcbi.1007614 (PMC7259805; doi:10.1371/journal.pcbi.1007614)
Supplement: S1 Text — (PDF) [file pcbi.1007614.s001.pdf]

S1 Text.
Variational latent Gaussian processes

Let $\mathbf{x}_t$ be the L-dimensional latent factors at time t, $\boldsymbol{\alpha}_n$ be the weights on latent factors of each neuron, $\boldsymbol{\beta}_n$ be the weights on covariates of each neuron, and $\mathbf{h}_{t,n}$ be the covariate of each neuron at time t. Under conditional independence, the joint distribution (data likelihood) of N neurons is given by

$$p(y_{1\ldots T,1\ldots N} \mid \mathbf{x}_{1\ldots T}, \boldsymbol{\alpha}_{1\ldots N}, \boldsymbol{\beta}_{1\ldots N}) = \prod_{t=1}^{T}\prod_{n=1}^{N} p(y_{t,n} \mid \mathbf{x}_t, \mathbf{h}_{t,n}, \boldsymbol{\alpha}_n, \boldsymbol{\beta}_n). \tag{S1}$$

Assuming Gaussian process (GP) framework for flexible prior design of each dimension $x_l(t)$ independently:

$$x_l(t) \sim \mathcal{GP}(\mu_l, \kappa_l) \tag{S2}$$

where $\mu_l(t)$, and $\kappa_l(t,s)$ are mean and covariance functions. Under discrete time, we have

$$p(\mathbf{x}_l) = \mathcal{N}(\mathbf{x}_l \mid \mathbf{0}, \mathbf{K}_l), \qquad l = 1, \ldots, L. \tag{S3}$$

We use the squared exponential covariance function for general smoothness over time,

$$\operatorname{cov}(x_{t,l}, x_{s,l}) = \sigma_l^2 \exp(-\omega_l (t-s)^2). \tag{S4}$$

where σ_l and ω_l are the magnitude and inverse time scale of the latent factors.

The posterior distribution over the latent factors

$$p(\mathbf{x}_{1\ldots L} \mid \mathbf{y}_{1\ldots N}) = \frac{p(\mathbf{y}_{1\ldots N} \mid \mathbf{x}_{1\ldots L}) p(\mathbf{x}_{1\ldots L})}{p(\mathbf{y}_{1\ldots N})}, \tag{S5}$$

is not tractable under a point process likelihood. Consequently, we utilize variational inference searching for an approximate distribution $q(\mathbf{x})$ to $p(\mathbf{x} \mid \mathbf{y})$. Then we rewrite the data likelihood

$$\begin{aligned}
&\log p(\mathbf{y}_{1\ldots N}) \\
&= \mathcal{E}_q[\log p(\mathbf{y}_{1\ldots N})] \\
&= \mathcal{E}_q\left[\log \frac{p(\mathbf{y}_{1\ldots N}, \mathbf{x}_{1\ldots L})}{q(\mathbf{x}_{1\ldots L})} \cdot \frac{q(\mathbf{x}_{1\ldots L})}{p(\mathbf{x}_{1\ldots L} \mid \mathbf{y}_{1\ldots N})}\right] \\
&= \underbrace{\mathcal{E}_q\left[\log \frac{p(\mathbf{y}_{1\ldots N}, \mathbf{x}_{1\ldots L})}{q(\mathbf{x}_{1\ldots L})}\right]}_{\mathcal{L}(q)} + \underbrace{\mathcal{E}_q\left[\log \frac{q(\mathbf{x}_{1\ldots L})}{p(\mathbf{x}_{1\ldots L} \mid \mathbf{y}_{1\ldots N})}\right]}_{D_{\mathrm{KL}}(q\|p)},
\end{aligned} \tag{S6}$$

where $\mathcal{E}_q$ denotes an expectation over $q(\mathbf{x})$, and $D_{\mathrm{KL}}(q\|p)$ is the Kullback-Leibler divergence. We maximize the lower bound $\mathcal{L}(q)$, also known as the Evidence Lower BOund (ELBO) to find the optimal q.

Assuming q factorizes into Gaussian distributions of each latent dimension

$$q(\mathbf{x}_{1\ldots L}) = \prod_{l=1}^{L} \mathcal{N}(\mathbf{x}_l \mid \boldsymbol{\mu}_l, \boldsymbol{\Sigma}_l). \tag{S7}$$

the evidence lower bound (ELBO) to be optimized is as follows

$$\begin{aligned}\mathcal{L}(q) &= \sum_{t=1}^{T}\sum_{n=1}^{N}\mathcal{E}_q[\log p(y_{t,n} \mid \mathbf{x}_t, \mathbf{h}_{t,n}, \boldsymbol{\alpha}_n, \boldsymbol{\beta}_n)] - \sum_{l=1}^{L}\mathcal{E}_q\left[\log\frac{q(\mathbf{x}_{1\ldots L} \mid \boldsymbol{\mu}_l, \boldsymbol{\Sigma}_l)}{p(\mathbf{x}_{1\ldots L} \mid \mathbf{K}_l)}\right] \\ &= \sum_{t=1}^{T}\sum_{n=1}^{N}[y_{t,n}(\boldsymbol{\alpha}_n^\top\boldsymbol{\mu}_t + \boldsymbol{\beta}_n^\top\mathbf{h}_{t,n}) - \exp(\boldsymbol{\alpha}_n^\top\boldsymbol{\mu}_t + \boldsymbol{\beta}_n^\top\mathbf{h}_{t,n} + \frac{1}{2}\boldsymbol{\alpha}_n^\top\boldsymbol{\Sigma}_t\boldsymbol{\alpha}_n)] \\ &\quad - \frac{1}{2}\sum_{l=1}^{L}[\boldsymbol{\mu}_l^\top\mathbf{K}_l^{-1}\boldsymbol{\mu}_l + \mathrm{tr}(\mathbf{K}_l^{-1}\boldsymbol{\Sigma}_l) - \log\det(\mathbf{K}_l^{-1}\boldsymbol{\Sigma}_l) - T].\end{aligned} \tag{S8}$$
