# Supplementary material for: Stimulus-choice (mis)alignment in primate area MT
Source: PLoS Comput Biol. 2020 May 18;16(5):e1007614. doi: 10.1371/journal.pcbi.1007614 (PMC7259805; doi:10.1371/journal.pcbi.1007614)
Supplement: S1 Fig — (PDF) [file pcbi.1007614.s002.pdf]

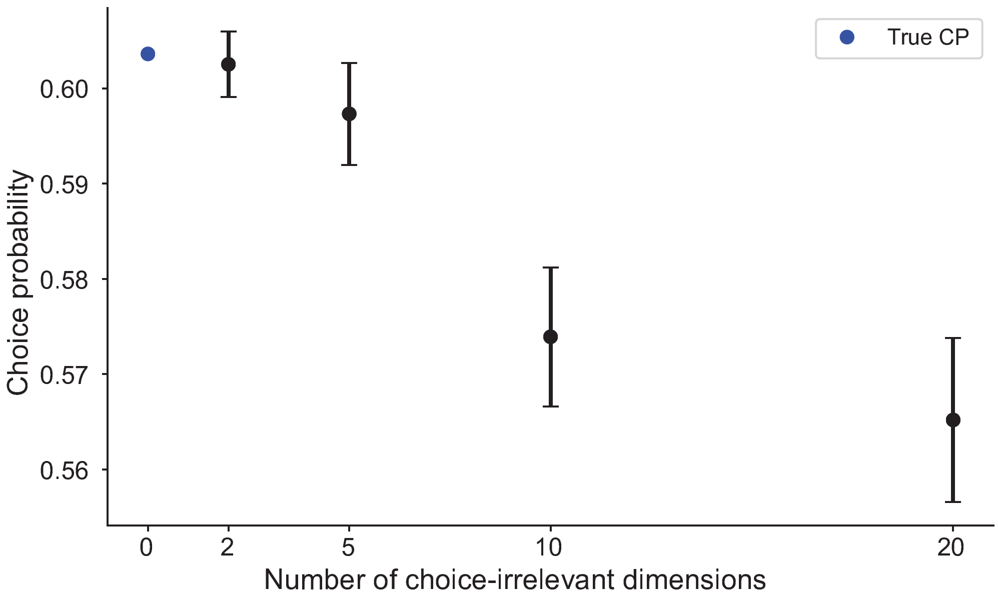

S1 Fig. Choice mapping does not inflate choice probability. Our choice mapping was cross-validated and the CPs were calculated on test sets. Hence CP cannot be inflated by overfitting. We confirmed that the higher dimensionality does not result in inflating CP (see Material and Methods) by the following experiment. We started with a 1-dimensional Gaussian sample drawn from $\mathcal{N}(0.2, 1)$ and $\mathcal{N}(-0.2, 1)$ randomly (size = 500 each). The choice probability of the 1-dimensional sample is 0.6 (blue). Then we add extra choice-irrelevant dimensions drawn from $\mathcal{N}(0, 1)$ independently and perform choice mapping as well. The resulting CPs (with s.e.m.) show that extra dimensions without choice information do not inflate CP.
