# Supplementary material for: Stimulus-choice (mis)alignment in primate area MT
Source: PLoS Comput Biol. 2020 May 18;16(5):e1007614. doi: 10.1371/journal.pcbi.1007614 (PMC7259805; doi:10.1371/journal.pcbi.1007614)
Supplement: S2 Fig — (PDF) [file pcbi.1007614.s003.pdf]

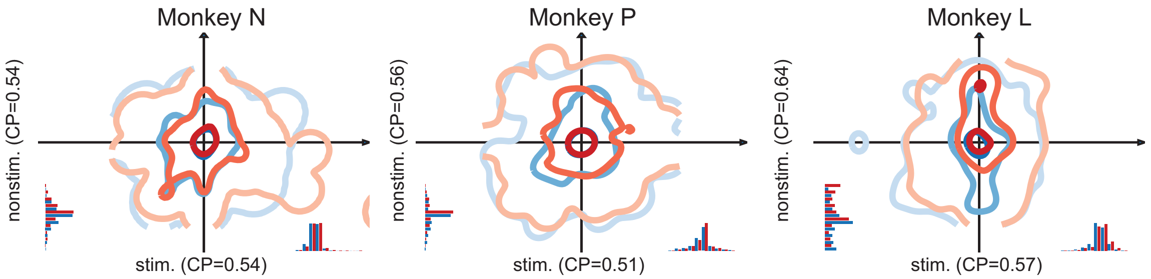

S2 Fig. (Pseudo frozen trials) Choice probabilities of latent factors for each monkey. To confirm that our procedure to select weak trials did not introduce a correlation between stimulus and choice, we repeated the choice probability analysis on the trials which we matched the hits and misses by randomly removing correct trials until the accuracy dropped to 50% for each session. We refer to the balanced set of trials as **pseudo frozen trials**. We performed the same analysis (see Materials and Methods) on the pseudo frozen trials. The results do not change the conclusion from the weak trials (S2 Fig and S3 Fig). Contours corresponds to 50%, 90%, 99% quantities of the choice-mapped stimulus and non-stimulus trial distributions (c.f. Fig. 1). The IN choice distribution (red-shade contours) is biased upward, indicating existence of the choice information in the non-stimulus axes. The pooled choice probability estimated using the choice-mapped stimulus-axis, non-stimulus-axes (the 3-dimensional subspace orthogonal to the stimulus-axis), and all 4 dimensions of the latent factors are 0.546, 0.591, and 0.621 respectively. The estimated population spike count choice probability is 0.627. For nested statistical tests of the corresponding regression models, see main text and Fig. 5.
