# Supplementary material for: Stimulus-choice (mis)alignment in primate area MT
Source: PLoS Comput Biol. 2020 May 18;16(5):e1007614. doi: 10.1371/journal.pcbi.1007614 (PMC7259805; doi:10.1371/journal.pcbi.1007614)
Supplement: S4 Fig — (PDF) [file pcbi.1007614.s005.pdf]

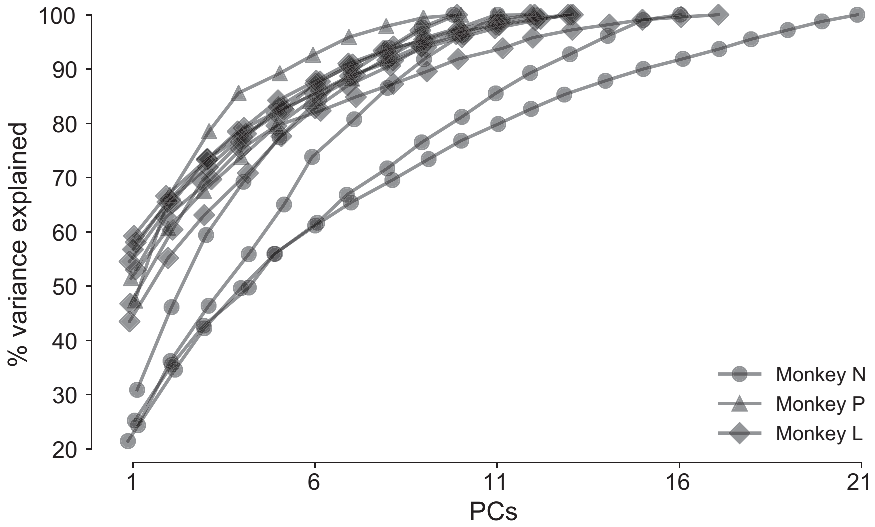

S4 Fig. Cumulative explaining power of principal components of raw spike trains. To compare with the linear method, we have performed PCA on the raw spike train binned at 100 ms (S4 Fig). The first 4 PCs couldn't capture the noise correlation well. Each line represent the cumulatively explained variance of one session. The shape of markers indicates the respective monkey.
