# Supplementary material for: Stimulus-choice (mis)alignment in primate area MT
Source: PLoS Comput Biol. 2020 May 18;16(5):e1007614. doi: 10.1371/journal.pcbi.1007614 (PMC7259805; doi:10.1371/journal.pcbi.1007614)
Supplement: S5 Fig — (PDF) [file pcbi.1007614.s006.pdf]

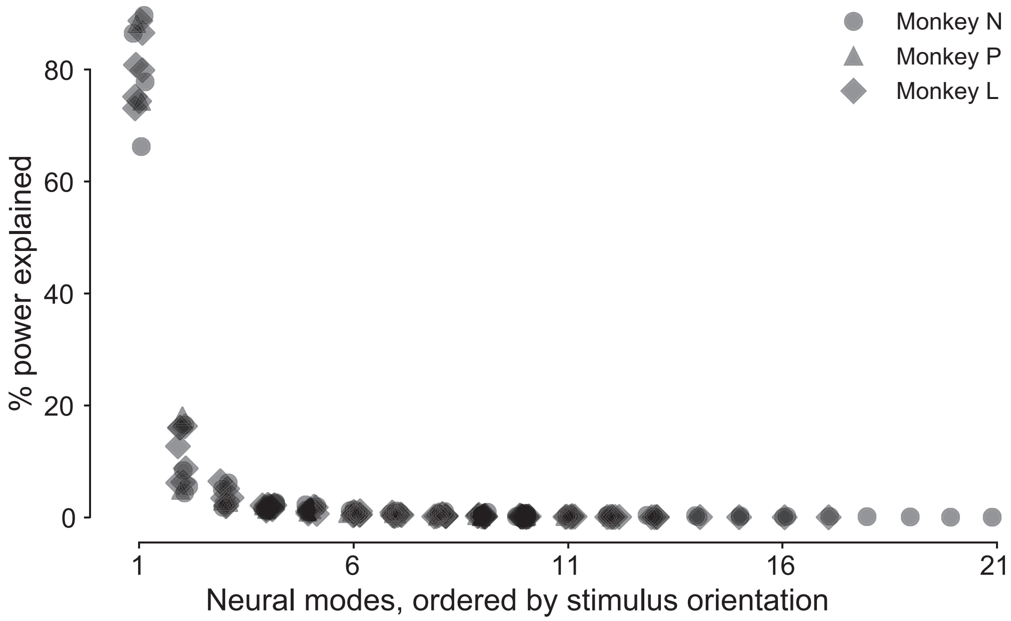

S5 Fig. Visual motion pulse information encoded in one dimension, which agrees with the vLGP result. The power of each factor that explains the variation contributed by the stimuli to the neural modes. Each marker indicates one session and the shape indicates the monkey.
