# Supplementary material for: Stimulus-choice (mis)alignment in primate area MT
Source: PLoS Comput Biol. 2020 May 18;16(5):e1007614. doi: 10.1371/journal.pcbi.1007614 (PMC7259805; doi:10.1371/journal.pcbi.1007614)
Supplement: S6 Fig — (PDF) [file pcbi.1007614.s007.pdf]

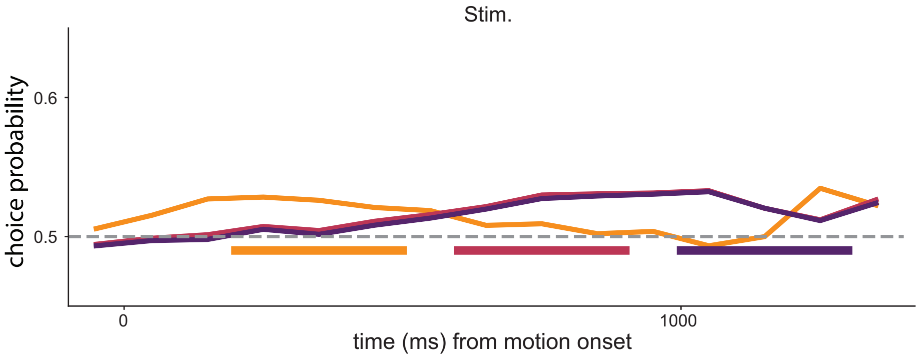

S6 Fig. (Raw spike count) Time course of choice probability in the stimulus subspace. The time courses of CP from the population spike count is consistent with the non-stimulus axis analysis. Decoders were fit to early (yellow), middle (red), and late (purple) periods (300 ms, marked by the colored bars) of non-stimulus latent factors to predict choice. We used the resulting weights of the decoders to perform choice-mapping on the whole time interval divided into 100 ms non-overlapping moving windows (aligned at the center). The colored curves correspond to the choice probability time course using the respective decoder.
