# Supplementary material for: Stimulus-choice (mis)alignment in primate area MT
Source: PLoS Comput Biol. 2020 May 18;16(5):e1007614. doi: 10.1371/journal.pcbi.1007614 (PMC7259805; doi:10.1371/journal.pcbi.1007614)
Supplement: Attachment — Submitted filename: 20200322_PLOSCB_MT_response_to_reviewers.pdf [file pcbi.1007614.s008.pdf]

We thank the reviewers for their careful reading of our manuscript and helpful comments. They led to substantial improvements to the manuscript which reflect significant additional analyses and text revisions. In the following we address all concerns point by point.

Reviewer #1: The authors applied a nonlinear statistical method to extract latent factors from MT neurons during a decision making task. Then, they analyzed the dimensionality of the stimulus and choice encoding axes. Overall, I found the paper to be interesting and generally sound. I have some technical concerns, as well as some confusing aspects of the figures. These concerns should be readily addressable.

1. A major claim of this study relates to feedback signals and choice. However the timeline of the task is not clear to me. Please provide a schematic that shows the timeframe of when the stimulus appears, each of the 7 motion pulses, the subject's response, and when the putative feedback signal is detected. Please also indicate in what time frames the spiking data is analyzed. On pg 11, line 300 you state that you decode using time windows up to 1300ms, but I don't have any reference for what those times mean. Additionally in Figure 3, the horizontal axis is labeled with "time" but no scale is ever given.

We apologize for this oversight. We have included a new figure to show the task structure. Throughout the manuscript, the 0 ms is the beginning of the 1st visual motion pulse. Each trial contained 7 consecutive motion pulses (each last for 150 ms) and the duration of visual stimuli was 1050 ms in total. The analysis was using the data within -100 ms ~ 1400 ms relative to the motion onset. For Fig. 7, we refer to the time window 1000 ms~1300 ms as the late period that was to estimate one of the 3 choice decoders (the other two are of early 200~500 ms and middle 600~900 ms periods) when we considered the feedback signal could appear (indicated in the figure as horizontal time bars). We have also added a time scale bar to indicate all traces without explicit time axis.

2. Fig2. The noise correlation matrices are confusing to me. Is each matrix for a single session? There are 2 sessions for monkey N, and 2 for monkey P, and 7 for monkey L? What is the point of this panel? Is it to show that the model captures the noise correlations? If so, please quantify it instead of visual comparison. If the point is that the noise correlations differ between sessions, again, please quantify it. With respect to concern (1), the timeframe for the latent trajectory goes out to 1500 ms, how does this relate to task structure?

We apologize for the confusing layout. We have updated the figure (now Fig. 3) to clarify the grouping of sessions and alignment to the visual stimuli. The panel of correlation matrices were meant to demonstrate the extracted single-trial latent factors were able to capture the noise correlations. As per reviewer’s suggestion, we have quantified the average explained power of the correlation matrices in the text. On average they were 0.952 (s.e.m. 0.007).

3. (Minor) Fig3. I believe you plotted the factors from three trials, one from each monkey. But the wording of the legend was confusing to me. You might want to change "Three example trials

from each monkey are shown" to "Three example trials, one from each monkey, are shown." Again here, what is the scale of time?

We apologize for the confusion. Fig. 3 shows the pulse-triggered averages of **three example sessions (not trial)**, one from each monkey. It visualizes the weights of pulses on the latent factors that were estimated from each session. We have changed the caption and added the time scale to the figure.

4. (Minor) Fig 3. Bottom Right, the label says "stimulus oriented factors" but I think it would be more accurate to say "latent factors, ordered by stimulus orientation" or something like that.

We have changed the label as the suggestion.

5. (Minor) Fig 3. What does each marker from each monkey mean? Is it the average across all trials from that session? Please clarify.

Each marker represents one session. The shape indicates the monkey. The colors are corresponding to the example session per monkey. We have updated the figure and legend to clarify this.

5. Fig 4. The text states "the IN choice distribution ... is biased upwards." Really? For Monkey L that looks true, but not Monkey N. Your main point is that the CPs on the non-stim axis are > 0.5. In your computations of the CPs for each set of subspaces, please report the variability across sessions and across monkeys.

We have calculated and reported the standard error (s.e.m.) of CPs across sessions of each monkey in the text. Now the text reads:

> The pooled choice probability estimated using the choice-mapped stimulus-axis, non-stimulus-axes (the 3-dimensional subspace orthogonal to the stimulus-axis), and all 4 dimensions of the MT latent trajectory are 0.546 (s.e.m. across sessions: Monkey N 0.013, Monkey P 0.007 and Monkey L 0.012), 0.591 (s.e.m. across sessions: Monkey N 0.009, Monkey P 0.020 and Monkey L 0.027), and 0.621 (s.e.m. across sessions: Monkey N 0.013, Monkey P 0.012, Monkey L 0.011) respectively.

6. Fig 4. How much does your result depend on your nonlinear encoding model? For example, could you arrive at the same conclusion by using a linear method to find the stimulus encoding dimension and computing the choice probability in that dimension vs other dimensions of the data? Please comment on the use of a nonlinear method, or provide a comparison with a linear method.

To compare with the linear method, we have done PCA on the raw spike train that was binned at 100 ms bin width. The result shows that the (cross-validated) explanatory power of the first 4

PCs to the noise correlations was -0.877 on average (s.e.m. 0.303). This suggests that the linear method couldn't capture the noise correlation with this number of factors. We also added the PCA cumulative power plot to the supplementary materials (Fig. S5).

7. I believe you included "weak stimulus" trials so you could have more data. When you did this you controlled the accuracy rate to be below 65%. I believe this means you did not match the number of hits and misses for these weak stimulus trials. Is it possible that this introduces a correlation between stimulus and choice? Please comment, or ideally, repeat the analysis by randomly removing hit trials until the accuracy rate is 50% (which would match the behavior on zero coherence trials).

As per reviewer's suggestion, we have selected subsets of 50% accuray by randomly removing hit trials and done analysis on those subsets. The results do not change the conclusion and are reported in supplementary materials (Fig. S3).

8. Please compare your methods and results for this recent paper, which appears to develop a similar method and comes to similar conclusions.
https://www.biorxiv.org/content/10.1101/808584v1.full.pdf

Although similar in spirit, our method differs greatly with the [Aoi et al 2019] approach in a critical manner. In [Aoi], they analyze the dynamics of the (conditional) mean firing rates, while we analyze **single trial** simultaneously recorded populations. They make several additional fundamental assumptions, (1) only the mean across the trials carry information, (2) the correlation between the neurons can be ignored, (3) the difference in the mean is being readout for the correct trials. In contrast, our analysis only analyzes the correlation (signal driven, choice drive, noise, etc) and disregards the individual neurons' private chatter. Only a single trial analysis can exploit the population level code, computation, and choice probability. We argue that our analysis is orthogonal to Aoi and coworker's approach. We have contrasted the relevant literature that analyzes only the mean activity (which is most of neuroscience) in the discussion section as follows:

> We use a Bayesian inference framework, vLGP [28], to demix the visual stimulus, the reported choice, and the trial-to-trial variability signals presented in the population activity. In contrast to other demixing methods [41,42], our approach does not require trial-averaging to obtain conditional mean firing rates nor a fixed trial structure necessary for the averaging. Analyzing the trial-averaged responses is convenient as simultaneous recording is not necessary, however, it comes with strong assumptions about the neural code: the noise correlation between neurons are ignored and only the differences in the conditional mean firing rates are assumed to carry useful information. Note that our analysis heavily relies on the marginal correlations (shared variations) present in the simultaneous recording. Moreover, only a single-trial analysis can reveal choice probability.

Reviewer #2: The authors find a low-dimensional representation of population recordings in MT while animals performed a sensory discrimination task. This low-dimensional representation defines the “stimulus-axis”. They also define different choice axis and find that those orthogonal to the stimulus-axis have a stronger correlation with animals’ choice than the one defined by the stimulus-axis. They claim that this finding provides evidence in favor of sub-optimal read-out with feedback as the origin of choice-related signals in sensory areas. This could be a useful strategy for transmitting feedback signals without harming the neural code.
The manuscript is an interesting piece of work and it represents a relevant contribution to the field. However, the clarity of the introduction, presented results, and methods, is not high and the accessibility could be improved in some sections. I also have additional concerns about specific points in the methodology and results and how they support some of the claimed conclusions, see below.

Major

1. The methods used to address the main scientific question of this study are sound and suited for it. However, a simpler and more straightforward approach to this question should be explored. The authors should compare the direction of two classifiers: one that has been trained on predicting stimulus identity and another that has been trained on predicting animal’s choice. Ideally, the choice classifier should be trained on non-evidence trials (or weak stimulus conditions where stimulus-driven activity has been regressed out). Finding similar results with this simpler analysis, would imply strong evidence in favor of the main finding of this study.

We thank the reviewer for this suggestion. We performed extra analysis using the raw spike counts to validate some of the main findings. As we reported in the manuscript, the population spike count choice probability is very close to the CP by the latents. We have further verified that the CP on the stimulus axis defined by the logistic regression weights from the raw spike counts to the stimulus is much smaller, consistent with the nonlinear latent factor results (stim: 0.53, nonstim: 0.61, see line 308). Furthermore, the time courses of CP from the population spike count is also consistent with the non-stimulus axis analysis (see Fig. 7 bottom and Fig. S6). We note that some of the co-authors of this manuscript have been actually working on in-depth analysis using linear / logistic regression methods including in [Yates 2020].

Yates, J. L., Katz, L. N., Levi, A. J., Pillow, J. W., & Huk, A. C. (2020). A simple linear readout of MT supports motion direction-discrimination performance. Journal of Neurophysiology, 123(2), 682–694.

Finally, we would like to note that PCA results (see #R1.6 for details) have failed to show a low-dimensional co-variability structure of the population, compared to our approach. The low-D non-stimulus subspace (or manifold) wouldn’t have been discovered if we used linear methods.

2. When describing Fig. 1 in the Introduction, the authors say (line 42): “In this space, ...”. This statement is slightly misleading because they are neglecting the effect of the covariance matrix

on the optimal read-out. If the population's covariance is not isotropic, the optimal read-out is (Gaussian approximation) proportional to the inverse covariance matrix times the "stimulus axis". This happens throughout the text, and given that this is central to the motivation of this work, the authors should clarify why they refer to the optimal read-out as parallel to the stimulus-axis and modify the text and figures accordingly.

Thank you for pointing out the subtleties. In our analysis, we define the optimal readout by training a classifier to readout, hence it takes the covariance structure into account to determine the optimal linear readout direction. The stimulus information can lie in more than 1 dimension in general as the reviewer pointed out and a nonlinear readout may be able to exploit such information. As we showed using regression analysis, stimulus information is mostly in 1D over time. This is important since our conclusion suggests a non-information limiting mechanism (i.e. the non-corrupting feedback). In the cartoon model of Fig 1, we have ignored the possible influence of population covariance for simplicity. However, we recognize that this might mislead the naive reader. Thus, we have modified the text to emphasize this subtlety. Now the relevant introduction paragraph reads:

> To visualize the space of hypotheses and how they can be distinguished, it is helpful to summarize the joint activity of a population of neurons with respect to the stimulus driven activity. Fig. 1 demonstrates this alignment conceptually and the effect of each type of choice models in this space. Specifically, for a population of only two neurons, the joint activity of the population can be represented as point clouds in a 2-dimensional space where each axis represents an individual neuron's activity (Fig. 1A). For a 1-dimensional stimulus (as is typically used in discrimination paradigms), different values of the stimulus (red and black) drives activity that falls along a 1-dimensional "stimulus axis". For simplicity, in this toy example, we assume there is no structure in the co-variability that can be exploited for decoding. In this case, increased variability along the stimulus axis (the so-called information limiting noise) will change the amount of information about the stimulus, while, importantly, variability orthogonal to the stimulus axis will not [19,22–25]. We call this variability direction the "non-stimulus axis" (Fig. 1A). In larger populations, the stimulus may reside in a subspace of higher dimension, however, we can use statistical classification methods to determine the stimulus subspace and non-stimulus subspaces in general.

In the discussion section we added:

> By linearly aligning the latent factors to the stimulus and behavioral choice, we were able to investigate how stimulus and choice are shared across neurons. Although we assumed a linear readout, note that the optimal nonlinear readout may change this interpretation [41–43].

3. The authors should clarify why the stimulus axis is defined upon the first variable of the 4D latent model. Couldn't it also be defined directly as the direction joining the mean of the two

distributions? Or even better, the direction defined by a classifier trained on decoding stimulus identity?

Yes, absolutely. Our 1st latent is NOT the factor that carries the most variance, but in fact the result of regression. Since the latent factors are subject to arbitrary rotation, we did perform linear regression to predict the latent factors by stimulus and then rotate the latent factors so that they are ordered by the stimulus-explaining power. In the text we say,

> The latent factors are subject to arbitrary rotation [26] which results in models with equivalent explanatory power. Hence, we rotated the latent factors for each session so that the effects of motion pulses are concentrated in decreasing order across dimensions (Fig. 4A).

4. The order the different panels of Fig. 1 appear in the text (Introduction) is a bit chaotic. It is also a bit unclear what exactly the different hypotheses are. Even though in the "Sensory and choice …" section it is mentioned again, the authors should be more explicit about the different hypothesis (i.e. i) …, ii) …, iii) …, etc.) and how they are related to Fig. 1 already in the Introduction. The panels should also be ordered in order of appearance in this section.

We have rewritten, itemized the hypotheses and ordered the text in the same order of the Fig. 1, and added a chart to Fig.1 to clarify the information flow.

5. The latent model validation seems to have been performed poorly. In Fig. 2B it is only visually shown how the structure of neuronal correlations is similar to those generated by the latent model. The authors should add a figure or table with the cross-validated explained variance of the model for all monkeys. Fig. 2B should also include a quantification of the similarity between the two matrices.

We have quantified the average explained power of the correlation matrices in the text. On average they were 0.952 (s.e.m. 0.007). See text 240-250 (See also reply to reviewer 1's 2nd comment.)

6. It is unclear why the number of latent variables is set to 4. The authors should specify why their criterion was based on the session with the largest number of neurons. Is it consistent across all monkeys? Is 4 the optimal number for only that session, for few of them, or for most of them?

The dimensionality 4 was determined by cross-validation on the session in which the largest number of MT units were recorded as we assumed that the population dynamics stably reside in similar manifolds across the sessions and monkeys while they were performing the same tasks. Unilaterally using 4 dimensions may not be optimal for each session, however, it should be high-enough to capture the variability (as seen in the noise correlation analysis). The cross-validation produces a more stable account for the dimensionality for larger sessions.

Hence, for a stable analysis across all sessions, we fixed the dimension to 4. We do not claim that this is optimal for analysis nor do we claim that the true dimensionality of MT population is 4. In fact, the statistically stable dimensionality is likely to be higher if we had access to a larger population recording. Overestimating the optimal dimension won't cause much harm, especially since our CP estimates are crossvalidated (see response to the next comment).

7. Comparing the influence of the stimulus-axis (1-dimensional) and the non-stimulus space (3-dimensional) is somehow unfair because 3 dimensions will in general be more informative than 1 dimension. It would be strong evidence in favor of the hypothesis of this study to show that the CP for the stimulus axis is smaller than the CP for each non-stimulus dimension evaluated individually. The authors should address this point, and in case of a negative result, explain how this can be consistent with their hypothesis.

Our estimation of CP is slightly non-traditional since we use cross-validation. In fact, we have seen severe overestimation of CP with previous methods naively extended to population choice probability. This prompted us to develop a more stable method. We have added text and a synthetic example (supplementary section 2 with figure S1) to clarify that the choice mapping does not inflate CP even with more dimensions in the method section. Now the text reads:

> In order to prevent inflation of choice probability due to high dimensionality (3D or more), we not only regularized the choice decoder to estimate the weights but also use the choice mapped values of the test set (pooled samples held-out by cross-validation). This approach guarantees that the overfitting of choice decoder will not result in overestimating the choice probability. The synthetic example (Fig. S1) also verified that adding choice-irrelevant dimensions does not inflate the choice probability.

8. Fig. 6 is confusing. Why is the stimulus-axis CP (time profile) not shown in this figure? The fact that curves purple and red peak later in the trial is expected because of the way the classifiers were trained. I am a bit skeptical that Fig. 6 shows indeed evidence in favor of the main finding of this study. Using these curves as well as the stimulus-axis CP time profile, the authors should be more clear on why Fig. 6 provides evidence in this regard.

We have added the stimulus-axis CP and neuron population CP time course to the figure. See also the newly added supplementary figure S6.

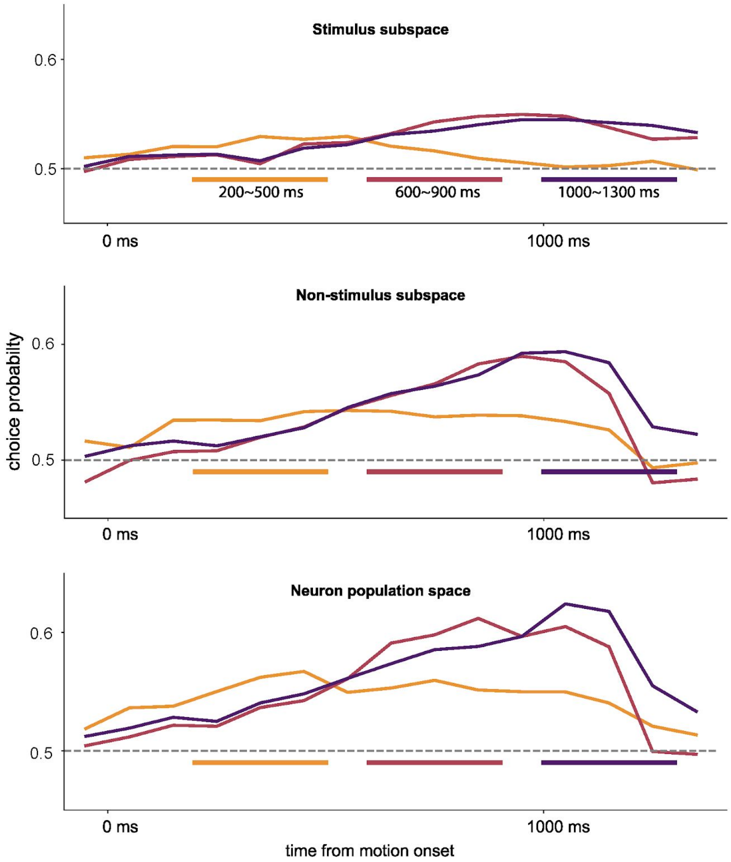

Minor

- In Fig. 1 it would be helpful to also depict how information is affected for each different scenario (panel). Please add some graphical clarification in this regard.

We have updated the figure and associated text to make it more clear.

- Please, add a paragraph where it is briefly explained how the feedback signals can affect CP. Readers might not be fully familiar with that literature and it would make the text easier to follow.

We have rewritten the paragraph of hypotheses in the intro section. How the feedback signals affect CP is explained in the entries (iii) and (iv).

- Even though the behavioral protocol is explained in ref. 24 and 29, it would improve the clarity and readability of the manuscript to describe the experiment in more detail in the methods section. The reader should be able to get a good picture of the dataset without the need to read refs. 24 and 29. For instance, the “Pulse-triggered average” section in the methods would be clearer with more detailed description of the stimulus presented to the monkeys.

We have added text and a figure to describe the experimental setup (Fig. 2).

- In the Methods section some of the equations are numbered and others are not. It would improve the clarity of the manuscript to have all numbered.

We have numbered all the displayed equations.

- In the "Single-trial latent dynamics of population" section (Methods), the vLGP model is poorly explained. For readers that are unfamiliar with variational inference it would be useful to have an explicit expression for the log-likelihood of the model as well as the ELBO term to be maximized. It should also be stated what term in the ELBO is going to be approximated by the equation in line 112.

We have added the detail as section 1 in the supplementary materials.

- There is a typo in line 118: "the" shouldn't be there.

Thank you for pointing it out. We have removed it.

- Labels and figures (everything) in Fig. 2 are too small and therefore very difficult to read. They should be made larger.

We have increased the font size.

- The "Pulse-triggered average" section is unclear. It should be re-written to make it easier to follow. In line 140, Beta hasn't been defined (is it a typo for W?).

Thank you for pointing out the typo. We have corrected beta to W. We extended the explanation for PTA as follows:

> To describe the temporal dependence of the latent factors on the motion pulses, we calculated the pulse-triggered average (PTA) for each of the seven pulses [27]. The PTAs are the regression coefficients that predict the change in latent states. Specifically, each PTA corresponding to one of the 7 motion pulses represents the modulation of latent factors by a unit visual motion (a single patch of Gabor drifting in one direction during a pulse), assuming a linear scaling with motion strength (see Materials and Methods).

- The term "frozen trials" is important for this study and is used throughout the manuscript. However, it is never properly defined. Please, add a sentence both in the methods and the results section defining the term.

We have updated the manuscript to briefly explain 'frozen trials' in the main text and methods.

- The last section of the Methods should be rewritten in a clearer way. Even though in the results section it is visited again, it is conceptually a fundamental part of the study and it should be more explicitly described already in the Methods. In particular, it is not well stated how are c_stimulus and c_nonstimulus calculated.

We have added the details of calculation. Now the text reads:

> the values of c_stimulus, c_non-stimulus and c_population were calculated by Eq. 9 with the response r (Eq. 7) and weights {β0, β} estimated via Eq. 8 of corresponding axes.

- Fig. 4 is difficult to understand. The authors should add a more detailed explanation on both the results section and the Figure caption. Specifically they should make sure they answer: what is the distribution plotted for each monkey? How does this figure relate to Fig. 1? How does each panel relate graphically to its shown CP? Also, text is difficult to read, the size should be increased.

Thank you for your suggestion. We now refer to Fig 1 (cartoon version). As you probably guessed, those three plots show the joint distributions (colored by choice) of choice-mapped stimulus (x-axis) and non-stimulus (y-axis) subspaces per monkey. The marginal distributions of each axis were also shown near the two axes along with respective CPs (also colored by choice). This figure was to show the visual discrepancy between the distributions of two choices giving the hints of CP. Besides we have increased the font size.

- For point 2 of the major revisions, Averbeck et al. Nat. Rev. Neuro. (2006) and Nogueira et al., J. of Neuro (2019) could be cited given their relevance to this topic.

Great point. We have cited relevant papers in the introduction.

> For simplicity, in this toy example, we assume there is no structure in the co-variability that can be exploited for decoding. In this case, increased variability along the stimulus axis (the so-called information limiting noise) will change the amount of information about the stimulus, while, importantly, variability orthogonal to the stimulus axis will not [19,22–25].

- In line 15, Kafashan et al. (2020) bioRxiv2020.01.10.902171; and Bartolo et al. J. of Neurosci. (2020) 2072-19 are also very relevant studies in this regard.

Thank you for the suggestion. We haven't considered directly quantifying the information-limiting noise in our data. However, the stimulus regressions show less than 5% explained variance, meaning there are a lot of variance in the latent factors that are not explained by the stimulus decoding, and CP < 0.6 shows that they are not well explained by the choice either. Our interpretation is that the information limiting correlation analysis through simple decoding may

be insufficient or even possibly misleading for quantifying the “accessible information” in our case. However, we feel that this is out of the scope of this paper, and we defer the detailed study of information limiting correlation to future work. We have cited the relevant literature in the discussion:

> By linearly aligning the latent factors to the stimulus and behavioral choice, we were able to investigate how stimulus and choice are shared across neurons. Although **we assumed a linear readout [41, 42]**, note that the optimal nonlinear readout may change this interpretation [43]. Within the space spanned by the latent factors, we found that the sensory task variable was primarily captured by a single latent factor, indicating that high-dimensional visual stimulus was represented in a low-dimensional, task-relevant manner across the MT population and time. **This stimulus-dimension was only partially explained by the stimulus, suggesting a presence of information-limiting noise [19, 22, 41, 42] that may strongly influence the animal’s choice**. However, the choice-correlated variability in the population of was mainly captured in latent subspace orthogonal to the stimulus-encoding dimension, which suggests that either the downstream decision circuit used suboptimal readout from MT response (Fig. 8, path 3) or the feedback from downstream circuit was non-corrupting (Fig. 8, path 4).
